# Manual and semi-automated approaches to MIBG myocardial scintigraphy in patients with Parkinson’s disease

**DOI:** 10.3389/fmed.2022.1073720

**Published:** 2022-12-02

**Authors:** Cecilia Boccalini, Giulia Carli, Emilia Giovanna Vanoli, Antoniangela Cocco, Alberto Albanese, Valentina Garibotto, Daniela Perani

**Affiliations:** ^1^School of Psychology, Vita-Salute San Raffaele University, Milan, Italy; ^2^In vivo Human Molecular and Structural Neuroimaging Unit, Division of Neuroscience, Scientific Institute for Research, Hospitalization and Healthcare (IRCCS) San Raffaele Scientific Institute, Milan, Italy; ^3^Laboratory of Neuroimaging and Innovative Molecular Tracers (NIMTlab), Geneva University Neurocenter and Faculty of Medicine, University of Geneva, Geneva, Switzerland; ^4^Department of Nuclear Medicine and Molecular Imaging, University of Groningen, University Medical Center Groningen, Groningen, Netherlands; ^5^Nuclear Medicine Unit, San Raffaele Hospital, Milan, Italy; ^6^Department of Neurology, Scientific Institute for Research, Hospitalization and Healthcare (IRCCS) Humanitas Research Hospital, Milan, Italy; ^7^Department of Neuroscience, Catholic University, Milan, Italy; ^8^Division of Nuclear Medicine and Molecular Imaging, Geneva University Hospitals, Geneva, Switzerland; ^9^Center for Biomedical Imaging, Geneva, Switzerland

**Keywords:** Parkinson’s disease, MIBG scintigraphy, H/M ratios, manual-drawing approach, semi-automatic fixed-size ROIs

## Abstract

**Objective:**

This study investigates the effects of manual and semi-automatic methods for assessing MIBG semi-quantitative indices in a clinical setting.

**Materials and methods:**

We included ^123^I-MIBG scans obtained in 35 patients with idiopathic Parkinson’s Disease. Early and late heart-to-mediastinum (H/M) ratios were calculated from ^123^I-MIBG images using regions of interest (ROIs) placed over the heart and the mediastinum. The ROIs were derived using two approaches: (i) manually drawn and (ii) semi-automatic fixed-size ROIs using anatomical landmarks. Expert, moderate-expert, and not expert raters applied the ROIs procedures and interpreted the ^123^I-MIBG images. We evaluated the inter and intra-rater agreements in assessing ^123^I-MIBG H/M ratios.

**Results:**

A moderate agreement in the raters’ classification of pathological and non-pathological scores emerged regarding early and late H/M ratio values (κ = 0.45 and 0.69 respectively), applying the manual method, while the early and late H/M ratios obtained with the semi-automatic method reached a good agreement among observers (κ = 0.78). Cohen-Kappa values revealed that the semi-automatic method improved the agreement between expert and inexpert raters: the agreement improved from a minimum of 0.29 (fair, for early H/M) and 0.69 (substantial, in late H/M) with the manual method, to 0.90 (perfect, in early H/M) and 0.87 (perfect, in late H/M) with the semi-automatic method.

**Conclusion:**

The use of the semi-automatic method improves the agreement among raters in classifying’ H/M ratios as pathological or non-pathological, namely for inexpert readers. These results have important implications for semi-quantitative assessment of ^123^I-MIBG images in clinical routine.

## Introduction

Parkinson’s disease (PD) and parkinsonisms are a spectrum of multisystem neurodegenerative diseases characterised by early involvement of the autonomic and enteric nervous systems (1). PD-related neuropathology (α-synuclein aggregates) is not restricted to the central nervous system but affects several organs and tissues (2, 3). The denervation of the autonomic nervous system can be assessed by ^123^I-metaiodobenzylguanidine (MIBG) scintigraphy (4), which can be clinically helpful as a supportive diagnostic tool (5). ^123^I-MIBG, a norepinephrine analogue (6), is taken up into the presynaptic cardiac sympathetic nerves by the norepinephrine uptake-1 transporter, and the amount of ^123^I-MIBG retention over several hours after administration reflects neuronal integrity (7). Thus, ^123^I-MIBG cardiac scintigraphy measures the postganglionic presynaptic cardiac sympathetic nerve endings of the noradrenergic nervous system. It was developed for the assessment of heart diseases (8). Subsequently, cardiac ^123^I-MIBG scintigraphy was clinically applied in the PD spectrum, showing a reduced cardiac uptake in the vast majority of patients (9), representing a sensitive test for this clinical condition. Therefore, positive results at ^123^I-MIBG scintigraphy are considered a supportive biomarker for PD detection (5) and differential diagnosis (10). ^123^I-MIBG scintigraphy can accurately distinguish Lewy Bodies (LB) related disorders [i.e., PD, Dementia with LB (DLB)] and non-LB related disorders (e.g., Alzheimer’s disease and progressive supranuclear palsy) (10).

The most used quantitative measurements of ^123^I-MIBG uptake are the heart-to-mediastinum (H/M) ratios calculated from early (15 min) and late (3–4 h) ^123^I-MIBG static planar images. The method used to obtain these parameters is mainly based on manually drawing cardiac regions of interest (ROIs). However, the position and size of the cardiac ROI, as well as the level of experience in drawing the ROIs on the planar ^123^I-MIBG images, might affect the final H/M ratio measures.

The effects of different methods to assess ^123^I-MIBG parameters for clinical interpretation should be carefully considered (11, 12). Therefore, this study aimed to compare two different methods assessing the H/M ratios in a series of PD patients, (i) the typical manual-drawing method and (ii) the fixed-size ROIs approach following Veltman’s guidelines for the standardisation of ^123^I-MIBG parameters. Veltman et al. (13) demonstrated good reproducibility of fixed-size mediastinal and cardiac ROIs in assessing H/M ratios in patients with heart failure; here, we wanted to extend these findings in PD. ^123^I-MIBG images of 35 PD patients were analysed by highly expert, moderate-expert, and inexpert raters. We evaluated the influences of cardiac ROI size and position and the impact of raters’ experience on assessing the H/M ratios.

## Methods

### Participants

The study included 35 patients fulfilling the PD clinical diagnostic criteria (5). All participants gave their written informed consent to the experimental procedure, as approved by the Ethical Committees of Scientific Institute for Research, Hospitalization and Healthcare (IRCCS) San Raffaele Hospital. The protocol conformed to the ethical standards of the Declaration of Helsinki for the protection of human subjects.

### ^123^I-metaiodobenzylguanidine scintigraphy data acquisition

^123^I-MIBG myocardial scintigraphy was performed at the Nuclear Medicine Unit, San Raffaele Hospital, using (NM/CT 670, Milwaukee, WI, United States) manufactured by GE Healthcare medical systems, according to the standard procedure (13). All patients have suspended medication and stopped eating any food that could influence ^123^I-MIBG myocardial uptake (14). A total of 111 MBq of ^123^I-MIBG was injected intravenously. ^123^I-MIBG planar images were obtained with a gamma camera in two temporal windows: 15 min after the injection (early-image) and 4 h after the injection (delayed-image). Static planar imaging was obtained with 256 × 256 matrix. Only planar images in thoracic anterior view were used for quantitative evaluation. All camera heads were equipped with low-energy high-resolution collimators and all images were acquired with a 15% energy window centred at the 159 keV photopeak of ^123^I. The image acquisition time was approximately 5 min. All planar images were analysed using Xeleris (Discovery NM/CT 670, GE Healthcare, Milwaukee, WI, United States) and PMOD software (PMOD Technologies Ltd.), for manual and semi-automatic methods respectively.

### ^123^I-metaiodobenzylguanidine scintigraphy planar image analyses

**FIGURE 1:**
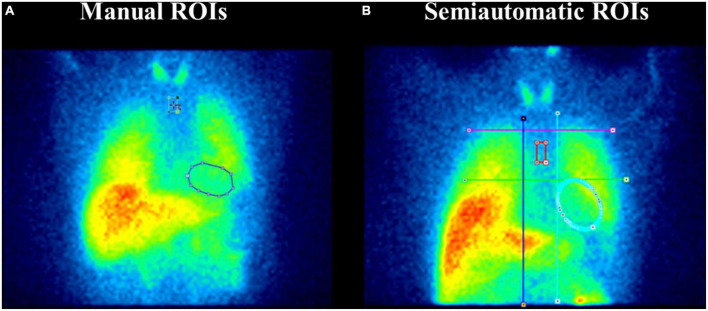
Manual and fixed-size regions of interest (ROIs) on planar ^123^I-metaiodobenzylguanidine (MIBG) images. Panel **(A)** depicts the manual ROIs method. A rectangular mediastinal ROI is used with unspecified size and located on planar MIBG images below the thyroid. The manual-drawn cardiac ROI is polygonal and includes the myocardium and the left ventricular cavity. Panel **(B)** represents the semi-automatic ROIs method. An “anatomical landmark square” is formed by the lung apexes (upper square border), the upper cardiac border (lower square border), and the medial contours of the lungs (medial square borders) to place fixed-size mediastinum and oval cardiac ROIs. Precisely, a fixed-size rectangular mediastinal ROI (13 × 20 pixel) and an oval cardiac ROI are placed on MIBG planar images. The mediastinal ROI is placed in the half of the “anatomical landmark square” formed by lungs apexes, the upper cardiac border, and the medial contours of the lung. The cardiac oval ROI (60 × 70 pixel) is placed in the lower-right quadrant, over the myocardium, including the left ventricular cavity.

Early and late H/M ratios were calculated from ^123^I-MIBG planar images using ROIs placed over the heart and the upper mediastinum. The ROIs were derived using two different approaches: manually drawn ROIs and semi-automatic fixed-size ROIs. In the first approach, a rectangular mediastinal ROI has been used with unspecified size. The cardiac ROI was a polygonal manually drawn ROI including the myocardium and the left ventricular cavity (Figure 1A). The manual method was performed on a Xeleris workstation, following the recommendations described in the proposal for standardisation by Flotats et al. (14).

For the semi-automatic method, we followed the guidelines of Veltman et al. (13) (Figure 1B). An “anatomical landmark square” was formed by the lung apexes (upper square border), the upper cardiac border (lower square border), and the medial contours of the lungs (medial square borders) to place fixed-size mediastinum and oval cardiac ROIs. Precisely, a fixed-size rectangular mediastinal ROI (13 × 20 pixel) and an oval cardiac ROI (60 × 70 pixel) were placed on ^123^I-MIBG planar images. The mediastinal ROI was placed in the half of the “anatomical landmark square” formed by lungs apexes, the upper cardiac border, and the medial contours of the lung. The cardiac oval ROI was placed in the lower-right quadrant, over the myocardium, including the left ventricular cavity. The use of fixed-size ROI, which the operators only need to move according to the anatomical landmark square, gives partial automation to the method. This semiautomatic method was processed using the PMOD software.

Three raters (expert, moderately-expert, and not expert) analysed the ^123^I-MIBG images using both procedures. Of note, the inexpert rater followed a brief training (2 h) for the manual drawing analysis. The other raters were experienced in the analysis of ^123^I-MIBG myocardial scintigraphy, with mean more than 5 and 10 years of experience, respectively. Anonymised data were analysed in a random order to avoid bias by order effects.

### Statistical analyses

The intra- and inter-rater agreements were tested to assess the reproducibility of the measured H/M ratios.

#### The inter-rater agreements

The intraclass correlation coefficients (ICC) were used to assess agreement among independent raters, considering the H/M ratios as a continuous variable. ICC values < 0.5, between 0.5 and 0.75, between 0.75 and 0.9, and greater than 0.90 are indicative of poor, moderate, good, and excellent reliability, respectively (15).

We applied previously published cut-off for pathological scans (<1.9 early ratios and <1.7 late ratios) to classify the H/M ratios into mutually exclusive categories (pathological and not) (16). Following this categorisation, we calculated the Fleiss’ kappa, κ to measure the inter-rater agreement between the raters (17, 18). The following classification was suggested for assessing the strength of agreement: κ < 0.20 poor, 0.21–0.40 fair, 0.41–0.60 moderate, 0.61–0.80 good, and 0.81–1.00 very good (19). Last, Cohen’s Kappa was also used to measure the agreement between two raters after classifying the H/M ratios as pathological or not. Cohen’s Kappa values 0.01–0.20, 0.21–0.40, 0.41–0.60, 0.61–0.80, and 0.81–1.00 were considered slight, fair, moderate, substantial, and perfect agreement, respectively (20). A Chi-square test was run to test differences in the proportion of patients classified as pathological or not by different raters.

#### The intra-rater agreements

The correlations between the H/M ratios obtained with the two methods by each rater were analysed by the Spearman correlation coefficients. The significance level was *p* < 0.001.

All statistical analyses were performed using the SPSS software package, version 26.0 (SPSS, Chicago, IL, United States).

## Results

A total of 35 PD patients [63.66 ± 9.74 years of age, 27 (77%) men] were included in the study. Table 1 reported the number of PD patients that fell into the category of “pathological sympathetic innervation,” according to the cut-off value of early and late H/M ratios, obtained with manual approach and semi-automatic approach for the three raters.

**TABLE 1:** Inter and intra-rater indices on ^123^I-metaiodobenzylguanidine (MIBG) parameters.

	Early H/M ratio		Delayed H/M ratio	
	Manual method	Semi-automatic method	Manual method	Semi-automatic method
**Inter-raters analysis**				
**Continue values**				
Expert (1)	1.49 ± 0.27	1.59 ± 0.27	1.41 ± 0.37	1.51 ± 0.35
Moderate- expert (2)	1.54 ± 0.28	1.53 ± 0.26	1.46 ± 0.35	1.44 ± 0.33
Inexpert (3)	1.65 ± 0.32	1.62 ± 0.28	1.56 ± 0.40	1.51 ± 0.36
ICC (95% CI)	0.94 (0.80–0.97)	0.95 (0.90–0.97)	0.95 (0.89–0.97)	0.97 (0.95–0.98)
**Categorical values** **(*n* pathological/35)**				
Expert (1)	32	28	24	20
Moderate-expert (2)	31	31	24	26
Inexpert (3)	26	28	20	23
Fleiss Kappa (95% CI)	0.45 (0.44–0.46)	0.78 (0.78–0.79)	0.69 (0.69–0.71)	0.78 (0.78–0.79)
Cohen Kappa 1–2	0.63	0.76	0.66	0.68
Cohen Kappa 1–3	0.29	0.90	0.69	0.87
Cohen Kappa 2–3	0.54	0.68	0.75	0.79
**Intra-rater analysis**				
Spearman coefficient 1	*p* = 0.92, *p* = 0.000		*p* = 0.92, *p* = 0.000	
Spearman coefficient 2	*p* = 0.86, *p* = 0.000		*p* = 0.93, *p* = 0.000	
Spearman coefficient 3	*p* = 0.91, *p* = 0.000		*p* = 0.91, *p* = 0.000	

### The inter-rater agreements results

The raters showed excellent reliability measured by ICC in both methods (i.e., manual and semi-automatic) (Table 1). Of note, the agreement among raters tends to be higher in the semi-automatic procedure (ICC = 0.95 and ICC = 0.97, early and delayed H/M ratio, respectively) compared to the manual one (ICC = 0.94 and ICC = 0.95, early and delayed H/M ratio, respectively) (Table 1).

Fleiss’ kappa showed a moderate agreement in the raters’ classification of normal and pathological scores regarding early H/M ratio values (κ = 0.45, *p* < 0.0005) by using the manual method. Instead, the agreement was good when the early H/M ratio was obtained with the semi-automatic method (κ = 0.78). Similarly, the late H/M ratios showed increased raters compliance moving from manual (κ = 0.69) to semi-automatic approaches (κ = 0.78). Of note, with the semi-automatic method, fewer PD patients fell under the category “non-pathological” for the inexpert and the moderate-expert raters (Table 1), with a more significant agreement among raters (Table 1). Specifically, the chi-square test found significant differences between the proportion of subjects classified as pathological or not by expert versus inexpert raters and moderate expert versus inexpert raters (*p* < 0.05) only with the manual method. The differences were not significant between raters when they applied the semi-automatic method (*p* > 0.05).

Cohen Kappa values revealed that applying a semi-automatic method instead of the manual one leads to an improvement in agreement, especially between expert and inexpert raters; the agreement moved from 0.29 (fair, in early H/M ratios) and 0.69 (substantial, in late H/M ratios) to 0.90 (perfect, in early H/M ratios) and 0.87 (perfect, late H/M ratios).

### The intra-rater agreements results

The Spearman correlation analysis showed a robust intra-rater agreement in the assessment of early and delayed H/M values (Spearman Coefficient > 0.80, *p* < 0.001).

## Discussion

This study investigated the effects of different methods assessing ^123^I-MIBG parameters on the clinical interpretation of ^123^I-MIBG images. To do this, we evaluated the reproducibility of delayed and early H/M ratios using two types of approach: (i) manual-drawing method and (ii) fixed-size ROIs approach following literature guidelines proposed for heart failure (13). Our results indicate that the use of fixed-size ROIs located following an “anatomical landmark square” (semi-automatic approach) improves the agreement among raters in classifying’ H/M ratios as PD pathological or non-pathological. Of note, the semi-automatic procedure produced a perfect agreement between experienced and inexperienced observers (Table 1).

The use of a rectangular mediastinal ROI with unspecified size and manually drawn cardiac ROI is commonly used (21). However, fixed-size ROIs are recommended in the proposal to standardise ^123^I-MIBG parameters, but they were validated in patients with heart failure and without comparing the semi-automatic method with the standard visual one (13). Our results support and extend these findings in PD. The raters showed good inter and intra-rater agreements in early and late H/M ratios (continue values) applying manual-drawn and semiautomatic methods. This finding suggests that both methods for the calculation of the H/M ratios benefit from excellent reproducibility. However, the semi-automatic procedure showed a higher percentage of raters’ consistency, assessing both early and late H/M ratios (Table 1).

The classification of the H/M ratios into pathological or not according to clinical cut-off (16) further highlighted the differences in the approaches. The agreement among raters in classifying patients into pathological or not, according to H/M ratios measures (categorical values) was higher in the semi-automatic procedure than in the manual one (Table 1). This variation might be explained by the fact that the expert and inexpert raters showed low agreement, especially in assessing early H/M ratio values using the manual method (Cohen Kappa 0.29). Instead, in the semi-automatic procedure, the consistency between the raters became perfect (Cohen Kappa 0.90). This improvement suggests that the semi-automatic procedure improves the interpretation of planar ^123^I-MIBG images in clinical settings.

This study has some limitations, particularly the small sample size and the lack of a control group. The data used in the present study have been acquired in the context of a protocol aiming to profile the dementia risk in PD with imaging biomarkers, where the acquisition of healthy controls was not planned. Moreover, we are aware that the superimposition of non-cardiac structures in the heart ROI might affect the ^123^I-MIBG planar measures. This bias is intrinsic to the planar image technique because of the lack of three-dimensional information. A volumetric single photon emission computed tomography (SPECT) acquisition protocol would help to overcome this limitation, because of three-dimensional information. ^123^I-MIBG-SPECT imaging can minimise the superimposition of the anatomic structures seen in two-dimensional planar images (e.g., lungs overlapping the heart) (22). We used the planar procedure in our study to obtain the ^123^I-MIBG images because the quantification of global cardiac innervation using volumetric SPECT is less established than planar ^123^I-MIBG imaging (23). This bias would equally affect the H/M ratios obtained with manual and semi-automatic ROI placement, most likely not impacting our study’s main result.

## Data availability statement

The raw data supporting the conclusions of this article will be made available by the authors, without undue reservation.

## Ethics statement

The studies involving human participants were reviewed and approved by the Ethical Committees of IRCCS San Raffaele Hospital. The patients/participants provided their written informed consent to participate in this study.

## Author contributions

CB and GC contributed to the conception and study design, statistical analysis, interpretation of results, and drafted the manuscript. EV contributed to the conception and study design and revised the manuscript. AC and AA provided clinical data and revised the manuscript. DP contributed to the study design, interpretation of results, and revision of the manuscript. VG contributed to the interpretation of results and revision of the manuscript. All authors read and approved the final manuscript.
