# Erratum: Manual and semi-automated approaches to MIBG myocardial scintigraphy in patients with Parkinson's disease

**DOI:** 10.3389/fmed.2023.1171820

**Published:** 2023-03-03

**Authors:** Frontiers Production Office

**Affiliations:** Frontiers Media SA, Lausanne, Switzerland

**Keywords:** Parkinson's disease, MIBG scintigraphy, H/M ratios, manual-drawing approach, semi-automatic fixed-size ROIs

An omission to the funding section of the original article was made in error. The following sentence has been added: “Open access funding was provided by the University of Geneva.”

The original article has been updated.

